# Toward a Categorization of Virus-ncRNA Interactions in the World of RNA to Disentangle the Tiny Secrets of Dengue Virus

**DOI:** 10.3390/v16050804

**Published:** 2024-05-18

**Authors:** Clara Isabel Bermudez-Santana, Juan Carlos Gallego-Gómez

**Affiliations:** 1 Computational and theoretical RNomics Group, Center of Excellence in Scientific Computing, Universidad Nacional de Colombia, Bogotá 111321, Colombia; 2 Grupo de Medicina de Traslación, Facultad de Medicina, Universidad de Antioquia, Medellín 050010, Colombia; carlos.gallego@udea.edu.co

**Keywords:** virus–host cell interactions, ncRNAs, microRNAs, immune response, sfRNAs, DENV

## Abstract

In recent years, the function of noncoding RNAs (ncRNAs) as regulatory molecules of cell physiology has begun to be better understood. Advances in viral molecular biology have shown that host ncRNAs, cellular factors, and virus-derived ncRNAs and their interplay are strongly disturbed during viral infections. Nevertheless, the folding of RNA virus genomes has also been identified as a critical factor in regulating canonical and non-canonical functions. Due to the influence of host ncRNAs and the structure of RNA viral genomes, complex molecular and cellular processes in infections are modulated. We propose three main categories to organize the current information about RNA–RNA interactions in some well-known human viruses. The first category shows examples of host ncRNAs associated with the immune response triggered in viral infections. Even though miRNAs introduce a standpoint, they are briefly presented to keep researchers moving forward in uncovering other RNAs. The second category outlines interactions between virus-host ncRNAs, while the third describes how the structure of the RNA viral genome serves as a scaffold for processing virus-derived RNAs. Our grouping may provide a comprehensive framework to classify ncRNA–host-cell interactions for emerging viruses and diseases. In this sense, we introduced them to organize DENV–host-cell interactions.

## 1. Introduction

A fraction of the pervasive transcription of the human genome includes noncoding RNAs, such as microRNA (miRNA), piwi-interacting RNA (piRNA), long noncoding RNA (lncRNA), circular RNA (circRNA), transfer RNA-derived stress-induced RNA (tiRNA), small nucleolar RNAs (snoRNA), and small nuclear RNAs (snRNAs) [1,2,3]. However, uncovering their role in viral infections is challenging, and only for miRNAs and lncRNAs has it been better understood. miRNAs bind mainly to the 3′ untranslated region (3′ UTR) of a target messenger RNA (mRNA) to regulate gene expression [3,4] through miRNA–mRNA (RNA–RNA) interactions, which end in mRNA degradation or translation regulation. LncRNAs are emerging regulators of multiple biological processes. They act through different interactions, such as protein–protein, RNA–RNA, and RNA–DNA, which lead to the formation of chromatin-modifying complexes on specific genome positions and/or to molecular scaffolds that modulate transcription.

Given that viruses have the ability to escape from the immune system and that miRNAs and lncRNAs are associated with functions in immunity and immune-mediated diseases [5,6,7], it was not entirely surprising to identify that the viruses encoded ncRNAs. In 1966, the accumulation of virus-associated RNAs (VA RNAs) was detected during human adenoviruses infection [8], and later, it was reported that VA RNAs were targeted by DICER for processing miRNAs or competing for them [9,10,11]. In 2004, the Epstein–Barr virus (EBV) was the first human virus in which miRNA coding capacity was discovered [12], the annotation of which has reached 44 to date [13]. EBV-encoded RNAs (EBERs), BamHI-A rightward transcripts (BARTs), snoRNAs, stable intronic sequence RNAs (sisRNAs), and circRNAs are part of the list of other EBV-derived expressed RNAs in a variety of cell types and tumors during EBV infection [14,15]. EBV is not the only example of a virus for which the genome is known to express distinct RNAs during viral infection that may potentially be associated with the immune response. In consequence, we focus our review on examples of human viruses of public health importance, for which pathogenicity is associated with viral noncoding RNAs that mainly target molecules of the immune response. We do not attempt to provide a comprehensive survey of all the viral-derived RNAs. Our recommendation for miRNAs is the following reviews: [2,3,16,17,18,19,20,21,22], and for lncRNAs, these publications [7,23,24].

In order to catalog the information reviewed, we propose three categories. The first describes human ncRNAs activated during viral infection, and these are related to immune response evasion [2,25,26]. The second introduces examples of viral ncRNAs that modify human ncRNA expression, and the last one describes the role of the virus genome shape on viral RNA expression. The categories presented in the first section follow the same order in the second and are to be used as a frame to organize the same sort of information reported for DENV. We focused on DENV because it produces high morbidity and mortality worldwide, with approximately 56,923 cases during the first half of 2020 in Colombia [27] while COVID-19 was spreading.

Currently, the paradigm for studying virus-host relationships is shifting because of the amazing information that the shape of the virus genome has provided about its role in interactions with the immune system. However, sustaining this for all viruses is challenging since additional studies, both in vitro and in vivo, are needed to further understand the many unknown mechanisms behind this. That is why many of the claims we make here have been supported by other authors who have also analyzed them in reviews or published similar points of view. These have, therefore, been framed here. Although not the focus of the review, we propose that the study of the shape of the viral genome and its function in processing viral RNAs may become key benchmarks for the development of RNA-based vaccines and antivirals [28,29]. Our system of cataloging the information we consider may be of great use in organizing the data on the interactions that other viruses have among other hosts in the future.

### 1.1. Category: Examples of Human-ncRNAs Associated with the Immune Response in Viral Infection

Human miRNAs are compromised under viral infections that influence the regulation of viral replication and pathogenesis [17]. However, other miRNAs are directly related to the immune response. hsa-miR-155 in macrophages helps control the inflammatory response [25]. Its modulation in microglial cells infected with Japanese Encephalitis virus (JEV) was reported to enhance CD45 expression, thereby reducing the pro-inflammatory cytokines, modulating Complement Factor H (CFH) expression, and suppressing JEV replication [30]. In addition, hsa-miR146a was up-regulated, thereby suppressing NF-κB activity and disrupting antiviral JAK-STAT signaling, as well as helping JEV to evade the cellular immune response [31]. Both miRNAs are also affected by Human Immunodeficiency virus 1 (HIV-1). In 2012, TLR3 and TLR4, but not other TLRs, were, for the first time, shown to induce increased levels of hsa-microRNA-155, the overexpression of which has a remarkable effect against HIV-1 [32]. Moreover, miR-146a has been proposed to inhibit HIV-1 infection in CD4+ T lymphocytes due to its capacity to activate and control CXCR4 expression in a pathway involving PLZF [33].

A family of miRNAs best conserved in mammals, miR34/449, is also associated with viral infections. They have been the subject of research, and the reviewed literature is available at [34]. In general, the family comprises six homologous genes: miR-34a, miR-34b, miR-34c, miR-449a, miR-449b, and miR-449c. How some of these miRNAs affect the immune system can be seen in the following examples. While under EBV infection, miR-34a is strongly induced by the enrichment of NF-κB on its promoter [35]. A library of known human miRNA mimics was screened by Smith et al. in 2017 to find out whether miRNAs have an effect on the replication of dengue virus (DENV), West Nile virus (WNV), and JEV. They discovered that hsa-miR-34 increased the ability of infected cells to respond to infection through the interferon-based innate immune pathway [36]. Several other examples include the case of hepatitis C virus (HCV) modifying the expression of hsa-miRNA-449a and hsa-miRNA-107, which could activate the IL-6-mediated signaling cascade and induce HCV-mediated inflammatory responses, as well as fibrosis [37]. More recently, when studying the changes in miRNAs expression in the acute and chronic phases of HIV infection, the general expression of miRNAs in the plasma of patients (except for hsa-miR-122-5p) was found. In patients with acute HIV, they notably identified a strong innate immune activation via the up-regulation of miRNA and the early activation of angiogenesis [38].

In addition to miRNAs, lncRNAs have recently gained more attention as their role in the functioning of the immune response is better understood [7,22,24,39,40,41,42]. In order to initiate this topic, we wish to start with a virus that compromises the immune system in multiple facets, such as HIV, and for which lncRNAs are also implicated during its infection and latency. NRON lncRNA, for example, contributes to the latency of HIV [43]. In addition, the antiviral lncRNA NEAT1 (nuclear-enriched abundant transcripts) was found to be down-regulated upon the activation of resting peripheral blood mononuclear cells and purified CD4+ T cells [44]. This research was performed using HIV-infected donor blood. In addition, lincRNA-p21 is down-regulated following viral infection, resulting in a cascade of processes that ultimately evade apoptosis in macrophages [45]. An lncRNA with multiple positive regulatory targets is the lncRNA LINC00173, which regulates interferon gamma (IFN-γ), C-C motif chemokine ligand 3 (CCL3), and C-X-C motif chemokine ligand 8 (CXCL8) [46]. One long noncoding RNA interacting with NF-βB (NKILA) inhibits HIV replication and reactivation by suppressing the initiation of long terminal repeat (LTR)-driven transcription (LTR) [47]. Moreover, MALAT1 promotes HIV-1 transcription and infection.

In other infections caused by Japanese encephalitis virus (JEV), Rabies Lyssavirus (RV), Herpes Simplex virus (HSV), and Influenza A virus (IAV), NEAT1 has been found to significantly increase, which, as a result, represses the transcription of interleukin IL-8 signaling [48]. Remarkably, the human cellular barriers that SAR-CoV2 crossed also affected the expression of lncRNAs [49,50,51]. Both NEAT1 and MALAT1 were also found to be differentially expressed in patients with severe COVID-19 [52]. WAKMAR2, EGOT, EPB41L4A-AS1, and ENSG00000271646 lncRNAs were proposed as being potentially involved in cytokine signaling [53].

Other non-canonical ncRNAs are also compromised under viral infections and were reviewed in [54]. h-snoRNAs and h-piRNAs are altered in mouse lungs under SARS-CoV infection [2,55], although further research is required to confirm whether they can affect the immune response. Within A549 cells and mouse lungs, viral NS1 protein from Influenza A virus up-regulates h-vault RNAs, promoting viral replication by suppressing protein kinase R (PKR activation) and, consequently, inhibiting the IFN response [56]. Moreover, viral miniRNAs or v-mvRNAs processed by influenza A polymerase during infection also regulate interferon β, as they function as an antagonist of RIG-I [57]. Rather specifically, v-MiR-HA-3p suppresses the poly(rC)-binding protein PCB2, which is an essential component of innate antiviral immunity [58].

### 1.2. Category: Viral ncRNAs–Human ncRNAs Interactions

Compared to the previous category, v-ncRNA–h-ncRNA interactions are less documented, perhaps because of a lack of resolution in the experimental methods or because this interaction has evolved only in some viruses and some host cells. In any case, large-scale experimental efforts will be needed to understand whether their function is based on v-RNA—h-RNA interactions or on competition for biogenesis machinery, the regulators of transcription factors, or combinations thereof. At present, it is not easy to find experimental methods to measure and validate the existence of such interactions between ncRNAs in living systems. However, some of them have been followed by two existing methods, one specifically in viral infection and another in mouse models and human cell lines, where Cyrano lncRNA promotes the destruction of mature miR-7 [59]. Here, we present the result in the context of herpesvirus infection. Herpesviruses use their own ncRNAs during latency to control apoptosis and immune response in host cells [22,60]. *Herpesvirus saimiri* (HVS), a virus circulating in New World primates, expresses seven Sm class snRNAs called HSURs. In 2019, Gorbea et al. developed a method called individual nucleotide resolution RNA-RNA interaction by using crosslinking and capture (iRICC) to identify these interactions in vivo. Together, they discovered that HSUR2 recruits host miRNAs miR-142-3p and miR-16 to mRNAs in apoptosis in marmoset T cells in addition to HSUR2-mRNA interactions [61]. They then tested (in HeLa cells) the binding of miR-27 to HSUR1 to induce miRNA degradation and the binding of miR-16 by HSUR2 to host target mRNAs [62].

Under herpesvirus infection, it is reported that the expression of both the h-miRNAs and v-miRNAs of herpesviruses are altered in oral human keratinocytes [63]. The changes in expression of hsa-miRNA-155, -630, -943, and -489 are associated with changes in the expression of miR-K12-3-3-3p in Kaposi’s Sarcoma-Associated Herpesvirus (KSHV), miR-H1 of HSV1, and miR-UL-70-3p in Human Cytomegalovirus (HCMV) [21,64]. A further example is observed in HIV-1 infection, in which HIV1-miR-H could affect the expression of hsa-miR-149 [65]. Nevertheless, the mechanism of these RNA-RNA interactions remains to be understood.

Novel data to uncover the role of miRNAs in host defense against Hepatitis C virus (HCV) and Ebola virus (EBV) infections reveal that liver-specific microRNA-122 (miR-122) stimulates HCV internal ribosome entry site (IRES)-dependent translation due to RNA–RNA interactions, as well as host miRNA expression [66,67,68]. It was recently suggested, via a co-expression analysis, that lncRNA–mRNA interactions enhance the antiviral immune response during acute chikungunya fever in the whole blood of pediatric patients [69].

### 1.3. Category: Shape of the RNA Genome and the Processing of Viral RNAs

The viral genome structure is not static. In fact, it can vary, giving rise to a space of folding structures. Most of these novel conformations are a fundamental part of the architecture and functionalities of the viral genome, documented primarily for RNA viruses [70,71]. Certain forms are potential precursors of ncRNAs or can become targets for antiviral drugs. Such regions function as a specific structural conformation that the virus adopts during its life cycle, as has been studied in the 5′ and 3′ UTRs of flaviviruses [72,73] and HIV-1 [74] or in enteroviruses [75]. A marked increase in research has focused on many of these subregions, which are platforms for complex forms of the viral genome, although the experimental verification of their potential products is not always easy. For example, the remarkable plasticity of the RNA virus genome to fold is a well-known viral replicative property. It also controls the half-life of its mRNAs to guide the co-localization of the virus in the host cell cytoplasm. These emergent folds may be targeted by cellular machinery [70,72] to process ncRNAs in some viruses (as in flaviviruses [76]) or to fit into the network of hairpin structures required for virion assembly in bacteriophage MS [77].

The interactions seen at the genomic level in different RNA viruses have been studied to physically characterize the architecture of the viral genomes using atomic force microscopy (AFM) [78] and high-throughput selective 20-hydroxyl acylation analyzed by primer extension (SHAPE) [70]. SHAPE identifies the conserved and nonconserved conformations in the coding or intergenic regions between HIV-1 and lentivirus SIVmac239 [74]. Several other structured RNA regions have been predicted in viruses with double-sense RNA genomes. These include the *Arenaviridae* and *Tospoviridae* families [79]. Together with algorithms and computational approaches, these methods have been used to identify regulatory motifs called regulatory RNA elements (RREs) that are involved in processes such as (1) replication, (2) protein synthesis, especially for those viruses that have IRES, (3) control of the half-life of viral mRNAs, (4) location in the cytoplasm, (5) protein binding sites, (6) RNA–RNA interactions, and (7) other less common molecular processes observed in influenza virus types A, B, and C which control the splicing of viral RNA by using overlapping motifs in segments where their genome is organized [80].

**Figure 1:**
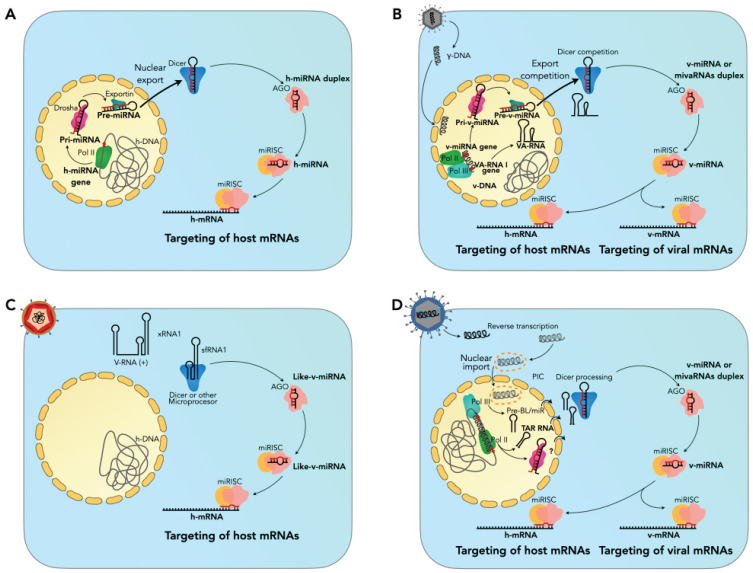
Schematic of three different possible scenarios for processing miRNAs, miRNA-type, and ncRNAs for viruses and host cells. Canonical cell miRNA biogenesis is schematized. Independently of miRNA locus activation, in (**A**), the main steps of this canonical processing in a generic host cell are shown based on Shapiro’s description [83]. Once RNApol-II transcribes the miRNA locus, its product is a precursor of miRNAs (pri-miRNA) that DROSHA will process into a pre-miRNA, as exported by exportin to the cytoplasm, to further proceed and be processed as a duplex RNA by the cytoplasmic endonuclease DICER. AGO will later recognize the mature miRNA to guide miRISC to a target messenger RNA. The processing of small RNAs from DNA genome viruses is presented in (**B**). There, VA RNAs are shown as precursors of v-miRNAs [10]. (**C**) Canonical processing of sfRNAs to produce small RNAs in RNA viruses with cytoplasmic replication, as in flaviviruses [84,85,86,87]. In (**D**), the possible biosynthesis of ncRNAs for retroviruses is shown. TAR RNAs are confirmed by [88], as well as BLV-miRNAs with activity in the RISC complex [89].

In addition to the critical functions of RREs, other folded regions in the vicinity of UTRs can be targeted by the small ncRNA processing machinery. This phenomenon uses the components of the miRNA processing pathway as a guide, coupled with the RNA-induced silencing complex (miRISC) to regulate target h-mRNAs [81]. Depending on the type of genome and where viral replication occurs, the biogenesis of viral miRNAs shares either partial or non-canonical mechanisms with h-miRNAs [76,82]. Although miRNA biogenesis is established in the host cell, the signals that activate a h-miRNA locus have yet to be discovered or clearly understood. However, once RNA pol II transcribes the miRNA locus, its product is a precursor of miRNAs (pri-miRNA) that DROSHA will export into a pre-miRNA by exporting it to the cytoplasm for further processing as a duplex RNA by the cytoplasmic endonuclease DICER. AGO will later recognize the mature miRNA to guide miRISC to a target messenger RNA. Figure 1 is an illustration of the differential processing of ncRNAs in viruses of public health importance, for which the model is most likely to have been tested. Figure 1A shows the major steps of this canonical processing in the host cell, as described by Shapiro et al., 2010 [83]. Unfortunately, the biogenesis of viral miRNAs and other viral ncRNAs is difficult to establish in the host due to their strong dependence on cellular machinery that, even within the same host cells, relies on a sophisticated regulatory network for orchestration that is difficult to trace.

For viruses with DNA genomes and nuclear replication, the biogenesis of v-miRNAs is remarkably similar to that of the host, as shown in Figure 1B. For example, host RNA polymerase II (RNA pol-II) transcribes the viral miRNA genome locus, which is processed by DROSHA from pri-viRNA to pre-viRNA before being exported to the cytoplasm for final processing by DICER. This type of mechanism is widespread in herpesviruses, papillomaviruses, hepadnaviruses, and polyomaviruses [21]. Finally, v-miRNAs can target h-mRNAs or their v-mRNAs. Two typical examples of this regulation occur during EBV infection: (1) the v-miR-BART2 targets the viral BALF5 polymerase and, thus, other h-mRNAs, and (2) miR-BART6-5p targets the DICER mRNA [12,90]. However, with an intermediate template derived from VA RNAs, another non-canonical pathway of v-miRNA processing also originates from DICER adenoviruses. Two VA RNAs are encoded by the adenovirus genome: VA-RNA I and VA-RNA II [10]. VA RNAs function against both long (interferon-induced) and short (RNAi-induced) dsRNAs by binding and inactivating two key enzymes, protein kinase R PKR and DICER. After being transcribed by RNA pol-II, a percentage of VA RNA molecules are processed into mivaRNAs (VA RNA-derived miRNAs) [10,11,91].

RNA genome viruses process RNAs according to the polarity of their genome (positive or negative strand) and where they replicate (cytoplasm or nucleus). The biogenesis of v-RNAs can vary and depends on whether the canonical machinery associated with h-miRNA processing is used. The influenza A virus RNA genome with nuclear replication produces mini viral RNAs (mvRNAs) of variable size, ranging from 56 to 125 nt in length, and other cases of mvRNAs smaller than 56 and larger than 125nt, which are independently processed by the h-RNAi machinery. They are the products of viral replication dysregulation and of viral RNA polymerase acting on dsRNA (double-stranded RNA) structures composed of the negative strand of the genome and its complementary RNA. These v-mvRNAs lead to the so-called “cytokine storm”, which is considered to be a factor associated with the Spanish pandemic flu caused by the influenza A strain 1 (H1N1) [57]. However, it remains to be determined whether their functions are an underlying event or a “macro” event or whether both are independent events. A more complex idea for the explanation of this miRNA-like RNA processing may be controversial and difficult to prove. It is proposed that after DROSHA translocation from the nucleus to the cytoplasm, this nuclease might process sfRNAs, producing an intermediate product that could be processed in a second step by an unknown cytoplasmic processor [76,82]. This type of translocation of DROSHA to the cytoplasm has only been demonstrated during infection with Sindbis cytoplasmic virus (SINV). In this case, a pri-miRNA type is processed by both DROSHA and DICER [92].

What is more commonly known, however, is that small viral RNAs can be processed into forms that differ from the canonical hairpin-like precursor structure of the host miRNAs. This processing is a fundamental step in viral infection cycles because some viruses recruit different components of the host cell biosynthetic machinery of miRNAs or because of the emerging inhibition of host ncRNAs biosynthesis under viral infection [93], including, perhaps, the functional sequestration of h-miRNAs [94]. These RNAs have been proposed as molecular markers in the design and selection of antiviral therapeutic targets [95] because of their interference with the RNA machinery. However, many questions remain regarding the understanding of their biogenesis. It is hoped that this will be aided by experimental validation [21]. For example, it has been proposed that KUN-mir-1 encoded by the WNV genome is the product of the DICER-dependent but RNApol II- and DROSHA-independent processing of the 3′UTR regions of the flaviviral genome [16,96]. This was first detected in the Kunjin strain of WNV (WNV(KUN)) derived from 3′SL [97].

Retroviruses with a single-stranded RNA genome and positive polarity use intermediate DNA for replication in the nucleus. These viruses exhibit biogenesis by means of canonical or non-canonical miRNAs [16]. However, the canonical pathways are still under debate because it is unclear whether they can generate the cleavage of the retroviral genome. Despite this uncertainty, other non-canonical pathways are better documented for this group. For example, the retrovirus (BLV) encodes hairpin-like forms that are transcribed by RNA pol III and processed by DICER in the cytoplasm [88]. However, they are independent of DROSHA nuclear processing, as seen in BLV-miR-B4 [89]. In HIV infection, the biogenesis of miRNAs is not fully understood, and for HIV1-miR-H [65], there are still some discrepancies [21]. However, miR-TAR-3p is processed by DICER from a hairpin-like structure presented in the precursor of trans-acting responsive TAR (TAR RNA). It is believed that miRNA processing occurs due to the pausing and premature termination of transcription by RNA pol II, the product of which is subsequently processed by DICER and coupled to AGO2 [88]. These three scenarios described for the processing of retrovirus-derived miRNAs are schematized in Figure 1D. TAR and the Rev response element (RRE) have recently been investigated for the development of anti-HIV therapeutics via targeted stabilization [98].

Subgenomic RNAs (sgRNAs) have been discovered in SARS-CoV-2 infection and are distinct from flaviviral sfRNAs in terms of biogenesis and function. This virus uses a unique replication mechanism called discontinuous transcription to generate sgRNA [99,100]. Other RNAs associated with viral infection-induced respiratory stress are the circRNAs encoded by MERS-CoV, SARS-CoV-1, and SARS-CoV-2. These circRNAs have also been identified and characterized [101]. Xhao et al., 2023, predicted 15 precursors for SARS-CoV-2-encoded miRNAs (CvmiRNAs), including 20 mature CvmiRNAs, in which CvmiR-2 was successfully detected by quantitative analysis in the serum and nasal swab samples from COVID-19 patients. The target gene prediction analysis suggested that CvmiR-2 may be involved in the regulation of the immune response, muscle pain, and/or neurological disorders in COVID-19 patients [102]. In 2018, Liu et al. reported for the first time the existence of complemented palindromic small RNAs (cpsRNAs) [103], which have not been previously reported for SAR-CoV-2.

Finally, the association of h-tRNAs with viral infections is undoubtedly one of the most surprising examples. tRNAs can be processed into small fragments known as tRFs, currently also known as tiRNAs [3], in addition to their conspicuous organization in eukaryotes [104,105]. tiRNAs can directly or indirectly influence RSV replication [106], viral gene expression during HCV infection [107], and antiviral immunity. These activities are proposed to resemble the anti-HIV activity of the cellular protein Schlafen 11 (Slfn11), which depends on a tRNA pool [54,108,109]. They also increase in chronic infections with Hepatitis B virus (HBV) or Hepatitis C virus (HCV) [110].

Table 1 summarizes the evidence for RNAs processed from viruses associated with the various human outbreaks discussed previously.

## 2. A Tropical Case: Dengue Virus (DENV)

The dengue virus is one of the most important infectious agents causing disease in the tropics. Found in more than 128 countries and with approximately 4 billion people at risk [120], it is considered the causative agent of one of the world’s most common diseases, dengue fever. It is caused by the four serotypes (DENV1-4) of the virus. Along with Chikungunya virus (CHIKV) and Zika virus (ZIKV), it is known as an arbovirus. It has spread rapidly throughout the world, with large-scale outbreaks in areas of the Western Hemisphere [121]. In 1635, there were suspicions of DENV-like outbreaks in Martinique and Guadeloupe, and in 1699, in Panama (or earlier) [122]. However, the endemic transmission of DENV-1 and DENV-2 in humans was not documented until after a surveillance program in Nigeria in 1964 [123]. Although several aspects have precipitated the emergence of arboviruses, in general, human impact on biodiversity and the alteration of natural ecosystems are the most relevant aspects [124,125].

The Dengvaxia^®^ vaccines, known as CYD-TDV and QDENGA^®^, are the only vaccines available for DENV. Dengvaxia^®^ has been the subject of much concern because it has been shown to cause severe disease in young, naive patients after natural infection [126]; however, the results of new clinical trials need to be reviewed in [127,128,129] to consider this vaccine as part of an integrated control strategy against endemic dengue [128]. Takeda’s next-generation DENV vaccine, QDENGA^®^, is still under evaluation [130,131,132]. This DENV vaccine is based on a live attenuated serotype 2 virus that is the genetic backbone for all four dengue virus serotypes and is designed to provide protection against each of these serotypes [133]. QDENGA^®^ has demonstrated long-term efficacy and safety against all four DENV serotypes in previously exposed individuals and against DENV-1 and DENV-2 in DENV-naive individuals, according to 5-year results from a phase 3 trial [132]. However, it is not yet clear how it works in travelers and is still being studied [130,131,134].

As the use of these vaccines is not yet conclusive, much effort should continue to be put into the search for strategies to control DENV and mosquito-borne viruses. These arboviruses, as well as other viruses such as EBOV, HIV, and SARS, share two characteristics, both functionally and structurally. The first is that they have RNA-type genomes, and the second is that they encode nonstructural proteins that act as antagonists of interferon and the immune response [135,136]. Traditionally, their interactions with host cells have been cellular factors, assuming only the role of the coding genome. Nevertheless, the function of the RNA genome as a noncoding RNA genome has gained increasing attention in the last decade and has contributed to the understanding of complex co-evolutionary processes between viruses and host cells. [137]. However, the role of secondary RNA structures in viral biology is mainly known in canonical processes such as viral replication [138]. In order to provide the context mentioned in the introduction for the organization of these RNA–RNA interactions, the categories introduced in a general way in the previous section for viruses of importance to human health will be presented below, with a focus on dengue virus.

### 2.1. Category: hsa-ncRNAs Associated with the Immune Response during DENV Infection

Since 2013, it has become clear that DENV disrupts the host RNA-mediated mechanism by regulating hsa-miR-625-3p, -767-5p, and -1200 [139,140] or directly by acting on hsa-miRNAs that target the human mRNAs of genes associated with the antiviral response [141]. For example, hsa-miRNA-146a acts as a proviral factor by targeting the TRAF6 gene. As a result, it inhibits the IFNβ-mediated defense system [142]. In 2014, further expression changes in hsa-miR-223, -281, -30e*, -146, -150, and let-7e-5p were reported. In particular, miR-150 and miR-30e * were found to be directly associated with regulating immune response [143,144,145,146,147]. In 2015, it was discovered that the up-regulation of let-7c regulates the mRNA of BACH1, which encodes a protein that is associated with anti-inflammatory and antioxidant processes [148]. Later, in 2017, members of the miR-34 family, canonically implicated in mediating the interferon response, were also found to be regulators of the viral replication of DENV and other flaviviruses such as West Nile virus (WNV) and JEV [36]. Concurrently, on primary human macrophages, miR-3614-5p was up-regulated and is proposed to be a down-regulator of the infectious capacity of DENV since the target is the RNA editing enzyme associated with the immune response (ADAR1) [149].

Other changes in hsa-miRNA expression have been studied, but it remains inconclusive whether they directly affect the immune response. For example, hsa-miR-223 inhibits dengue virus replication by negatively regulating the expression of stamina protein 1 (STMN1), which is known to regulate microtubule filaments in endothelial cell assays (EAhy926) [145]. Other studies detected the up-regulation of hsa-miRNA-625-3p, -767-5p, -1200, and -299-3p in blood samples from DENV-infected and uninfected patients [150]. In 2016, the human miRNAs hsa-miR-21-5p, 146a-5p, -590-5p, -188-5p, and -15-3p were also highlighted as non-invasive molecular markers of DENV infection [151].

Studies in the HeLa cell line (ATCC) have shown that miR-424 inhibits DENV2 infection by repressing the translation of three E3 ubiquitin ligases. DENV2 infection strongly induces the expression of SIAH1, which leads to the binding of SIAH1 and the ubiquitination of the TLR signaling adaptor protein MyD88. Thus, as a result of the regulation of miR-424, they suggest that there could be an additional mechanism by which DENV2 disrupts TLR signaling-activated cellular defenses through the induction of negative regulators of immune signaling [152]. By using a DENV-2 infection system based on the Huh-7 cell line, the function of miR-155 was investigated. It was found that through the induction of heme oxygenase-1-mediated antiviral interferon responses, this miRNA inhibits DENV replication [153]. In the same model, the role of has-let-7c was previously demonstrated [148]. In DENV-2-infected peripheral blood mononuclear cells (PBMCs) isolated from healthy individuals, miR-150, hsa-let-7e, and miR-146a were shown to regulate IL-8 expression [154].

Later, in 2021, Rossi et al. investigated the expression levels of 754 microRNAs during active DENV infection in a human monocyte cell model [155]. They found that hsa-miR-30a was up-regulated. Since hsa-miRNA-30e* has been reported as a suppressor of DENV replication by promoting NF-κB-dependent IFN production [147], the authors proposed that a similar mechanism could work in monocytes during DENV infection by hsa-miR-30a exerting the same action. However, the authors stated that this process needs to be confirmed. In the same study, two microRNAs—hsa-miR-548c-5p and hsa-miR-548d-5p—which were associated with innate immune responses in GO analysis, were reported to be down-regulated [155]. To summarize, the current function of human miRNAs in promoting or inhibiting DENV is shown in Table 2. We refer only to those reported in the context of the purpose described in this category.

We have mainly investigated the role of miRNAs in the immune response because it has traditionally been assumed that the pathogenesis of DENV is due to an exaggerated inflammatory response. However, another perspective could also explain the disease. For example, by evaluating changes in the miRNA expression in endothelial cells, we reported changes in the expression levels of miRNAs in endothelial microvascular cells in conditioned media after DENV infection. We propose a variety of biological processes that are activated and are mainly related to the migration of endothelial cells, a phenomenon that is called microvascular remodeling [159]. These results are consistent with an epithelial-mesenchymal transition, as we have recently hypothesized [160]. In particular, we reported that three miRNAs were up-regulated after DENV infection: hsa-miR-98-5p, -485-3p, and -4498. Interestingly, hsa-miR-98-5p induces mesenchymal–epithelial transition [161]. There is experimental evidence that senescence increases the susceptibility of infected endothelial cells [160], although many studies indicate that changes in microRNA expression occur as an inflammatory response after DENV infection [162,163].

Finally, we have also reported human tiRNAs as targets regulated by DENV infection [164]. DENV2-infected and uninfected HMEC-1 cells were used to evaluate small RNA expression. We found differentially expressed small RNAs derived from tRNAs previously known as tRF-3′ProTGG, tRF-3′ProAGG, tRF-3′GluCTC, and tRF-5′ValACC. Our results are consistent with the fact that tRFs substantially increase in chronic liver infections induced by chronic infection with HBV or HCV [110].

### 2.2. Category: hsa-ncRNA–DENV RNA Interactions

There is a need to better document the expected v-ncRNA–h-ncRNA interactions in the pathogenesis of DENV infection and, in general, for all viruses. However, when h-miRNAs interact with the UTRs of the DENV genome, other RNA–RNA interactions occur. For example, in 2014, the up-regulation of miR-281 under viral replication was discovered in C6/36 mosquito cells and in vivo in *Aedes albopictus*. It was shown that miR-281 may potentially target the 5′ UTR region of DENV-2. However, further research and experimental support are necessary for its validation [146]. Later, in 2017, the over-expression of miR-484 and miR-744 were found to be candidate inhibitors of DENV infection by acting on the 3′ UTRs of the four serotypes [156]. Other miRNAs have been proposed to directly target the DENV genome for the inhibition or promotion of DENV replication, such as Let-7a, which may target the NS1 region of DENV-2 [157,158].

### 2.3. Category: DENV Genome Shape and Its Role in vsRNA Processing

During flavivirus infection, abundant sources of small RNA are generated. Flaviviruses produce subgenomic RNA (sfRNA) antagonists of IFN-1 type [85]. They are products of the resistance of the RNA genome to enzymatic degradation due to the stalling of the of 5′-3′ exoribonuclease 1 (Xrn1) by the barrier imposed by the RNA structural forms—a kind of pseudo-knot—located in the flaviviral genomic subregions [85]. Interestingly, neither DENV nor ZIKA sfRNAs were first detected in 1997 in the brains of flavivirus-susceptible and -resistant mice after challenge with Murray Valley Encephalitis virus [165]. In 2008, by using West Nile virus as a model, the sfRNAs were discovered to be highly structured and nuclease-resistant RNA regions [84]. In 2016, Akiyama et al. demonstrated that ZIKV-infected monkeys and human epithelial cells, mouse neurons, and mosquito cells generate sfRNAs [166]. Particularly in ZIKV infections, sfRNAs lead to interactions with several host-derived RNA-binding proteins. These proteins modulate mRNA decay and splicing in infected human cells [86]. It has been shown that sfRNAs are an absolute requirement for ZIKV propagation [167]. In addition, sfRNAs have recently been associated with anti-apoptosis in ZIKV-infected *Aedes aegypti* [87]. In 2020, by using a new technique to dissect the determinants of sfRNA formation, SLI and SLII were found to have strong co-operation, supporting the higher-order organization of this region of the 3′ UTR region of DENV-2. However, further research and experimental support are necessary for its validation [146]. It has been shown that sfRNAs are an absolute requirement for ZIKV propagation [167].

ZIKV and DENV share a high degree of homology [167] in this region. ZIKV infects and targets human neural progenitor cells (hNPCs). If the mother is infected, ZIKV causes microcephaly in the fetus. In these cells and in brain organoids, ZIKV infection induces the abundant production of virus-derived small interfering RNAs (vsiRNAs) [116]. Recently, RNA pulldown and mass spectrometry assays demonstrated that abundant sfRNAs are produced in ZIKV-infected hNPCs. They found that sfRNAs interact with proteins that are part of the RNA-induced silencing complex (RISC) and with vsiRNAs. Furthermore, the 3′ stem loop (3′SL) of sfRNA [168] mediated the binding of RISC components and RNAi inhibition. In exceptional cases, sfRNAs can be processed downstream by canonical and non-canonical pathways to small miRNA-like RNAs, which are then associated with the RISC complex downstream. These pathways may or may not be dependent on the machinery that is associated with the classical processing of miRNAs in the host cell. This canonical pathway hypothesis is illustrated in Figure 1C: DICER targets the sfRNA to be processed into small miRNA-like RNAs [76].

**Figure 2:**
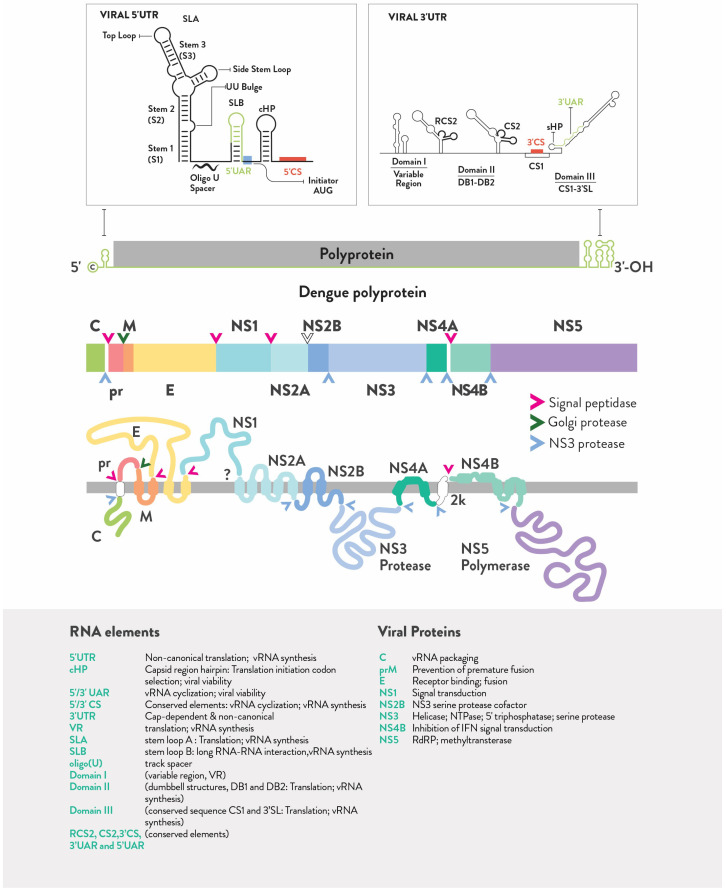
The major regions of the DENV genome are shown. At the top, we observed the projected zoom at the UTR regions. There, SLA, SLB, cHP, and domains I, II, and III fold into shapes with increased complexity on the UTR in 3D as described previously [169,170]. They were revealed using computational and experimental techniques, including folding prediction, probe analysis, and RNA interaction groups by mutational profiling (RING-maP). These UTR regions may be potential precursors for the processing of vsRNA-like miRNAs. RNA structures in the 3′ UTR are also resistant to Xrn1. The polyprotein is then plotted and the coding regions that encode the viral proteins are shown in their order. The signal for the peptidase, Golgi and NS3 proteases is also indicated by an arrow. The corresponding names are organized on the bottom boxes for both the RNA and protein parts of the DENV genome.

The major regions of the DENV genome are shown in Figure 2. It is interesting to note that SLA, SLB, cHP, and domains I, II, and III fold into shapes on the UTRs with increased complexity at the three-dimensional level, as described previously [169,170]. They have been revealed by using computational and experimental techniques, such as folding predictions, probe analysis, functional studies, SHAPE, and RNA interaction groups by mutational profiling (RING-maP) [170,171]. These UTR regions are considered to be potential precursors for processing vsRNA-like miRNAs. RNA structures resistant to Xrn1 are also found in the 3′ UTR, but other authors refer to them as flaviviral nuclease-resistant RNA (fNR) structures rather than sfRNAs. This name comes from 3D studies that revealed the existence of a three-way junction RNA fold instead of an SL structure in this subregion. In addition to the fact that other human nucleases associate with this subregion in the company of Xrn1, this proves that high energy is required to degrade them [172]. It was also reported that the size of the sfRNAs was different for each serotype: 430nt for DENV-1, 429nt for DENV-2 (strain 43 also produced two smaller sfRNAs of 180 and 270nt), 410nt for DENV-3, and 390nt for DENV-4 [173]. In 2015, Manokaran et al. combined experimental and phylogenetic analyses of DENV-2 using isolates from a 1994 outbreak in Puerto Rico and neighboring countries. They show that DENV subgenomic RNA binds to TRIM25 and suppresses interferon expression for epidemic fitness, demonstrating positive selection on DENV2 sfRNAs [174]. Later, in 2020, Syenina et al. added to these findings by revealing that five amino acid substitutions in the NS5 protein reduce viral genome RNA replication in favor of more abundant sfRNAs, which are then candidates to compete with the entire viral genome for packaging into infectious particles [175].

The existence of small viral RNAs from the DENV genome has been demonstrated in an experimental setting. However, understanding how this biogenesis occurs remains difficult to validate. If the DENV genome folds and takes on the shape of any RNA-like molecule, why not hypothesize that it could be a template target for processing? In 2014, Hussain and Asgari [96] studied the folding capacity of the structure of the 5′ UTR and 3′ UTR from the genome of DENV-2 (New Guinea strain). They identified six hairpin structures that were used along with deep sequencing for further analysis. vsRNA-5 was enriched in expression after infection from Aag2 cells and the C6/36 and Vero cell lines. Subsequently, they reported that the biogenesis of these DENV vsRNA-5-like miRNAs is DICER-1-dependent (involved in the biogenesis of miRNAs in insects) and AGO2-dependent (mediates the cleavage of pre-miRNA to produce mature vsRNA) but DROSHA-independent. Since the viral protein NS1 is a regulator of DENV replication, it has been proposed that vsRNA-5 regulates NS1. However, the enrichment of the vsRNA detected by Hussai in human cells (Huh7) infected with DENV-4 [176] could not be shown in another publication [176]. A curious example of the expression of these small RNAs is described as follows: no expression of DENV vsRNA-5 was detected in the vector *A. aegypti*, at least in Aga2 cells infected with DENV-2. Later, in 2015, Schirtzinger et al. characterized dengue virus-derived small RNAs in U4.4 mosquito and interferon-competent HuH-7 and in interferon-deficient Vero mammalian cells. In particular, they found 21-nucleotide RNAs mapped to the genomic hotspots of the DENV genome, with expression derived from the three cell lines. Although the length of these small RNAs varied from 12 to 36 nucleotides in mammalian cell lines, mainly from the positive sense genome, it was suggested that they were not a product of DICER processing [177]. Thus, this biogenesis remains a mystery. However, there was evidence that *Aedes* PIWI proteins produce DENV-derived small RNAs known as vpiRNAs (viral piwi RNAs), so other mechanisms may be involved in their production [117].

Despite the controversies regarding the type of small RNA that DENV can process and whether the context of expression is active on the host or in the vector, the experimental evidence shows that they can potentially perform in a context that may be cell-specific in at least some of the following four functions: (1) they may be part of the network regulating the expression of the DENV genome; (2) they may participate in the regulation of host cell genes [96]; (3) they may contribute to the evasion of the innate immune response and regulate the response to interferon type 1 (IFN-1); (4) they may regulate apoptosis latency and also lytic replication [19,118]. Although there is a long way to go to decipher biogenesis, a computational simulation performed by our group [178] detected putative microRNA-like structures that could be promising precursors of vsRNAs.

## 3. Discussion

In the last two decades, more and more interactions have been explored in which RNAs are essential, either because they are involved in RNA–RNA, RNA–protein, or protein–protein interactions, as explained by the following: (1) There is an antiviral immune response of the host cell against the viral infection; (2) the viral genome is composed of genes or subregions of the genome that encode proteins, ncRNAs, and other byproducts of the processing of the viral genome; (3) the expression of viral proteins affects the host expression of the host proteins; (4) viruses interact directly or indirectly with host ncRNAs, such as siRNAs, miRNAs, and lncRNAs, via viral proteins and sometimes their ncRNAs and vice versa; (5) depending on the host cell machinery, viruses have difficulty in completing their infection cycle. On the other hand, host cells will defend themselves by maintaining their homeostasis and resisting the establishment of viral infections [18]. Therefore, in order to include ncRNAs as key molecules, the canonical paradigm that has placed proteins at the top of the antiviral response must be modified [20].

Cells have antiviral mechanisms. However, the variety of living systems does not always share the mechanisms to activate them; the RNA interference (RNAi detected for the first time by Fire and Mello [179]) pathway via small interference RNAs (siRNAs) and RNA-dependent RNA polymerases (RdRPs) is mainly used in plants and invertebrates. In vertebrates, the innate immune system is activated by pathogen recognition receptors (PRRs). PRRs recognize foreign nucleic acids. This recognition triggers downstream signaling events that result in the synthesis of interferon (IFN) (discovered in 1957 [180,181]) and other cytokine genes. These cytokines stimulate the expression of more than 100 genes, for which a comprehensive network of 1,380 interactions between 265 TFs and 108 cytokine gene promoters has been constructed [83,182,183,184]. Due to the complex evolutionary history of RdRPs, which show frequent independent losses and functional liability in many animal lineages [81,83,185], this activation works independently of RdRPs in mammals. The importance of RdRPs is still under debate, but the competition between sfRNAs and vsiRNAs of ZIKA in human brain cells are examples of the role of host RNAi-based antiviral innate immunity. Regardless of the mechanism, proteins and RNA play an essential role in triggering the immune response, as illustrated by the examples shown here.

Our examples show how the viral genome influences the production of ncRNAs during infection. It is evident that some viruses share common strategies for ncRNA generation, whereas others do not. Consequently, others have evolved different mechanisms to produce different types of ncRNAs. Despite the machinery, the processing of small RNAs is likely to depend on the shape of the genome. How they are biogenized, however, remains poorly understood in many cases. For example, there are still many structured subregions on the viral genome that could be the target of positive selection to generate new regions that could become precursors of ncRNAs. Some examples have been found by our group for ZIKV and some betacoronaviruses [186,187,188]. However, it will take several more years to carefully integrate and validate these RNAs and their potential function as RNA mediators in silencing. For example, a rigorous experimental evaluation of whether filoviruses produce miRNAs was conducted by Prasad et al. [114] in 2020. Surprisingly, their work evaluated the potential for the abundant small RNAs derived from EBOV infection to function as v-miRNAs. By using molecular and immunological techniques, Prasad’s group showed that v-ncRNAs are independent of any biogenesis or function of host miRNA; that is, neither DICER, DROSHA, nor AGO are associated with their existence. Their results raise doubts as to whether these small RNAs correspond to miRNAs, despite their abundance in the progression of disease in EBOV infection. Therefore, these point to a complexity in the small RNA biogenesis, which is similar to the work carried out to map viral small RNAs to hotspots in the DENV genome [177]. Those small RNAs raise many questions about whether they are part or not of the virus-host relationship. These results should be of interest to the scientific community such that they can be the subject of monitoring. Our contention is that such an example could be an intermediate evolutionary step of a continuous progressive gain in biological function in an evolutionary scenario mediated by these curious small viral RNAs. This phenomenon could be evidence of slow “viral learning” in the silencing pathway of h-miRNAs.

It is important to note that, in some cases, host miRNAs can be antiviral, but in other cases they can increase viral replication [16,21] or stimulate translation [66]. These findings suggest that some viruses activate host miRNAs to facilitate or inhibit infection. Depending on the type of virus and cell target, it is important to study whether viral infection inhibits the maturation of h-miRNAs, stimulates their degradation, or acts as a factor promoting pathogenesis [16]. Evidence for the second category is in need of further investigation, as there is little information available. The examples mentioned here correspond to the expression of v-microRNAs: miR-K12-3-3p from KSHV, miR-H1 processed from the genome of HSV1, and the case of miR-UL-70-3p from HCMV inducing the expression of h-miRNAs -155, -630, -943, and -489 [21,64]. Only one case was mentioned for the retrovirus, HIV-1, where HIV1 miR-H acts as a regulator of h-miR-149 in T cells [65]. However, since some of the viral miRNAs interact with human miRNAs, which may be a byproduct of viral-host interaction evolution, this complex regulatory process needs to be better understood. Since human miR-281 could potentially target the DENV-2 5′ UTR region [146], miR-484 and miR-744 are candidates for targeting the 3′ UTRs [156] and let-7a to target the DENV-2 NS1 region [157,158], it would be essential to compare whether these effector functions are the same as the ones reported for hsa-miR-296-5p, which suppresses the replication of enterovirus EV71 by targeting the viral genome [189]. However, it would be essential to verify whether this effect, in both cases, is a consequence of the activation of hsa-miRNA, which could then potentially interact with viral product mRNAs to inhibit replication, or whether the RNA-miRNA interaction marks the viral genome for degradation.

Viral latency is another important issue that may be related to the function of viral ncRNAs. Although the viral latency for DENV was an old proposal [190] that lost interest in the scientific community in the following years, and in 1990, an authorized review showed emphatically that this possibility was rare [191]. However, the interaction of DENV-2 sfRNAs with the formation of stress granules (SGs) and the co-localization with proteins involved in SG formation under DENV2 infection, as well as their RNA silencing suppressor activity [118], reopened this discussion. Typically, viral latency is found in the *Herpesviridae* family, with specific genes producing this phenomenon, where the genome is incorporated into the host cell nucleus as an episome [60,61] and in HIV-1 latency periods [65]. Bryan Cullen of Durham University has been unraveling the complicated process of some of these viruses from the point of view of the relationship between the miRNAs and the proteins involved in the latency period. In the field of virology, the latency period to which this finding refers is very different from the latency period (a clinical concept) in the human host following infection by a mosquito bite. This concept has allowed us to understand the essential aspects of the natural history of DENV infection in both vertebrates and insects. Unfortunately, this critical aspect is only briefly addressed in this manuscript. Although there are indeed references from the biomedical field working with viruses where researchers consider latency as an aspect of their ecology [192,193], in the field of disease ecology, latency is not a central concept in the natural history of the disease. This is without mentioning that it is widely known that the circulation of viruses is part of the typical microbiome of many reservoirs, such as human beings.

Another point related to the importance of arbovirus-derived ncRNA could be related to its emergency in nature. The idea that arboviruses originated from arthropods is widely accepted [194]. The ancestral lineages may have originated from insect-specific viruses (ISVs) that were able to jump to a vertebrate host [195]. Since the majority of arboviruses have RNA genomes (genera *Bunyavirus, Rheovirus, Togavirus, Flavivirus,* and *Rhabdovirus*), this hypothesis is very plausible [196]. Because clones of viral RNA are highly abundant in arthropod transcriptomes, the absence of RNA polymerase correction is another important piece of evidence [197]. The fact that arbovirus cell lines are essential for the growth of these types of viruses, in contrast to mammalian cells, is further evidence. Therefore, although several researchers have recently searched for viral behavior differences in vectors or in hosts, we posit that noncoding RNAs could also be considered as molecules indicating host adaptation. The most relevant hipotesis analysis of this theoretical insight was also previously discussed in 2019 by Ohlund et al. [195]. They argued that the signaling cascades triggered by arbovirus adhesion/entry into midgut cells could induce an antiviral response by activating specific genes initiated by STAT phosphorylation. The release of dsRNAs from the viruses or from replication intermediates with dsRNA structures could then be processed by RISC complexes. In conclusion, arboviruses as emerging viruses pose a massive problem regarding the potential relationship between ISVs and ncRNAs and the understanding of their emergence.

Whether directly or indirectly, viruses and host ncRNAs can sometimes act as friends and sometimes as foes [16,20]. However, from an evolutionary point of view, this interpretation would be a manifestation of the trade-off between virulence (“enemies”) and transmission rate (“friends”). The consequence is viral fitness. To paraphrase the postulate of Paul Ewald [198], if viral yield increases, then the host cell allows more effective replication cycles using its ncRNAs. In the opposite situation, the interaction between viruses and host ncRNAs has a negative effect on the fitness of the virus. In this sense, current virus-host cell interactions are the result of many years of co-evolution, in which the participants struggle for survival. Therefore, in some cases, the virus–host interactions activate the immune response by employing strategies to evade the virus and maintain cellular integrity. In other viral infections, viruses evade this response.

The first part of the molecular biology of the virus was built on the paradigm that the protein is the only one responsible for controlling the virus-host relationship. However, the second part is about to be written with the discovery of the critical importance of ncRNAs. It has been suggested that ncRNAs, like the viral byproducts, may be less immunogenic than viral proteins and may become more critical in allowing viral infection to progress. In addition, they would be more efficient in the evasion of the host immune response and have an advantage in replication. They would also gain an advantage in replicating during infection, thereby increasing their fitness. These ideas are increasingly being used to study the virus-host relationship described here, as factors associated with these interactions can be studied in the development of vaccines and antivirals. Finally, ncRNAs may serve as possible biomarkers for the progression of the disease caused by DENV, as potential predictors of clinical risk, or as targets for antiviral therapy. In order to explore these possibilities, we suggest that the use of specialized methods should be investigated in depth.

At present, the following arboviruses are circulating in the tropics: ZIKV, CHIKV, and DENV, which are all known as emerging viruses. They are transmitted from reservoirs in wild animals to humans (a phenomenon known as zoonotic transmission): HIV has a chimpanzee reservoir, influenza A and its subtypes are transmitted from birds, and SARS-CoV, MERS-CoV, and EBOV are transmitted by bats. They have had a dramatic impact on the way we live and on public health [111,112]. As climate change progresses, the impact will increase dramatically [113]. When there is an impact on the environment, such as a change in the distribution and abundance of reservoirs—a change in the ecology—the virus populations change. This is the reason why viruses are not “latent”. They are not waiting for a species barrier jump. Viral emergence is simply an evolutionary consequence of ecological impact. The direction of emergence is a very complex phenomenon that depends on many variables, which are discussed for the evolution of the shape of sfRNAs in DENV [172].

Therefore, international efforts to contain emerging epidemics must rely on developing vaccines and antivirals and strengthening public health. However, SARS-CoV-2 is the only example where industrialized containment has been achieved. Vaccines and new treatments for these diseases have not always been successful despite immense scientific efforts to research and develop them. For example, for the Human Immunodeficiency Syndrome (AIDS) caused by HIV, the WHO indicated that 70 million infections, almost 35 million deaths, and approximately 37 million active cases had occurred by 2017 [65]. There is still no vaccine. Vaccines are only available for the Ebola and influenza viruses, which have been on the rise since the middle of the last century; Ervebo, or VSV-EBOV, is the advanced platform for EBOV [199]; the monovalent Audenz vaccine [200] for influenza A virus subtype H5N,1; and the multivalent vaccines for genera A and B, including subtypes H1N1 and H3N2 [201]. The outlook for arbovirus vaccines is less promising. The Dengvaxia ^®^ vaccine is in need of revision [127,128,129], and the QDENGA^®^ next-generation DENV vaccine is still under revision [130,131].

## 4. Conclusions and Perspectives

The three main categories proposed in this review allow us to organize the major studies of the 20th and 21st centuries on the role of ncRNAs in human viral infections. The examples highlighted here show how the human host factors that are usually associated with the innate immune response can also be targeted and regulated to co-operate with the expression of human ncRNAs during viral infection. Our categories provide a framework to reorganize insights into how human ncRNAs and viral ncRNAs are associated with the immune response under human viral infection, highlighting the special role that the shape of the viral genome has. Additionally, RNA viruses exhibit the plasticity of the genome shape in different stages of the infective cycle, and their role as possible precursors of small RNAs has been highlighted. The genome form can be understood as a characteristic of RNA viruses, mediating its infectious success by gaining the “coding” power of ncRNAs. Although most virus–host interactions are based on research derived from viral and host proteins, the studies of RNA viruses, host and viral ncRNAs, and RNAi together provide a strong and consistent set of processes that could now support the explanation of virus–host interactions in an RNA-dependent concept. However, a framework still needs to be integrated as a standard of molecular biology for viruses, which might follow the categories proposed here. Future investigations will need to take advantage of advances in RNA and RNA-RNA experimental approaches, next-generation sequencing, and synthetic biology to elucidate the role of RNA-RNA in complex interactions arising from viral infection.

## Figures and Tables

**Table 1:** List of viruses associated with outbreaks in recent decades. (**a**) shows the name of the virus and its taxonomic classification at the family level. (**b**) describes the type of viral genome. Note that the associated viruses are presented in genomes of the RNA type and sense (+ o − strand). The kind of ncRNA associated with viral infection reported in any of the three categories described here is presented in (**c**). (**d**) shows the epidemic [111,112,113].

a. Viruses/Family	b. Genome Type	c. Associated ncRNAs	d. Epidemy
Influenza A/*Orthomyxoviridae*	RNA single-strand negative-sense	h-vault RNAs [56]. v-mvRNAs [57].	2009–2010 “swine” or “North American” flu. Seasonal flu
Ebola (EBOV)/*Filoviridae*	RNA single-strand negative-sense	Small v-ncRNAs independent of miRNA biogenesis and synthesis [114]	Ebola epidemic in West Africa, 2013–2016
MERS-CoV/*Coronaviridae*	Positive-sense single-stranded RNA	circRNAs [101]	Currently active
SARS-CoV-1/*Coronaviridae*	Positive-sense single-stranded RNA	h-snoRNAs and h-piRNAs [55], h-lncRNAs [39], v-cpsRNAs [103], circRNAs [101]	2002–2003 Epidemy
SARS-CoV2/*Coronaviridae*	Positive-sense single-stranded RNA	sgRNAs, circRNAs, CvmiRNAs [99,100,101,102]	COVID-19 pandemic
HIV/*Retroviridae*	Reverse transcribed single-stranded RNA	h-lncRNAs [39]. v-miRNA [65,88]. h-miRNAs [33,65]	Currently active according to WHO [65]
ZIKV/*Flaviviridae*	Positive-sense single-stranded RNA	h-lncRNAs, [115], sfRNAs [86,87], vsiRNAs [116]	Zika fever, epidemic in Brazil, 2015
DENV/*Flaviviridae*	Positive-sense single-stranded RNA	DENV-vsRNA-5 [96], viral piwi RNAs [117], sfRNAs [85,118]	Annual epidemiological peaks. More than 3 million cases in Latin America [119]

**Table 2:** miRNAs reported to inhibit or promote DENV in two categories of interactions.

a. miRNA	b. Experimental Model	c. Target	d. Effects	References
**Category 1: hsa-ncRNAs associated with the immune response during DENV infection**				
miR-146a	PBMC	IL-8 expression	Promote	[154]
Let-7c	Huh-7, U937-DC-SIGN	BACH1	Inhibit	[148]
miR-155	Huh-7	Heme oxygenase-1-mediated antiviral interferon	Inhibit	[153]
miR-30e*	HeLa, U937, human monocyte cell model, PBMCs	NF-κB	Inhibit	[147,155]
miR-34 family	HeLa	IFN	Inhibit	[36]
miR-3,614-5p	Primary human macrophage	ADAR1 mRNA	Inhibit	[149]
miR-424	HeLa	E3 ubiquitin	Inhibit	[152]
**Category 2: hsa-ncRNA–DENV RNA interactions**				
miR-281	C6/36	DENV 5′UTR	Promote	[146]
miR-484, miR-744	Vero	DENV 3′UTR	Inhibit	[156]
Let-7a	Blood of humans and mice	NS1 sequence	Inhibit	[157,158]

## Data Availability

Not applicable.
